# Metabolomic breath landscape analysis unravels lipid biomarker candidates in patients with genetic and idiopathic Parkinson’s disease

**DOI:** 10.1038/s41531-025-01255-x

**Published:** 2026-01-10

**Authors:** Madiha Malik, Norbert Brüggemann, Tatiana Usnich, Max Borsche, Tobias Demetrowitsch, Björn-Hergen Laabs, Karin Schwarz, Peter Bauer, Katja Lohmann, Christine Klein, Thomas Kunze

**Affiliations:** 1 Department of Clinical Pharmacy, Institute of Pharmacy, Kiel University, Kiel, Germany; 2 Institute of Neurogenetics, University of Lübeck, Lübeck, Germany; 3 Department of Neurology, University Hospital Schleswig-Holstein, Lübeck, Germany; 4 Center of Brain, Behavior and Metabolism, University of Lübeck, Lübeck, Germany; 5 Institute of Human Nutrition and Food Science, Food Technology, Kiel University, Kiel, Germany; 6 Kiel Network of Analytical Spectroscopy and Mass Spectrometry, Kiel University, Kiel, Germany; 7 Institute of Medical Biometry and Statistics, University of Lübeck, Lübeck, Germany; 8 University Medical Center Göttingen, Göttingen, Germany; 9 Centogene, Rostock, Germany

**Keywords:** Biomarkers, Diseases, Neurology, Neuroscience

## Abstract

Parkinson’s disease (PD) is the fastest growing neurodegenerative disorder. The current lack of efficient early diagnostic tools necessitates novel approaches to biomarker discovery. We propose an untargeted metabolomics approach using non-invasive exhaled breath analysis. Breath samples, collected from 73 PD patients, encompassing both genetic (*LRRK2*: n = 12, *GBA1*: n = 35, *PRKN*: n = 6) and idiopathic PD (n = 20), 4 unaffected *LRRK2* pathogenic variant carriers, and 90 controls underwent extreme-resolution FT-ICR-MS analysis. Findings were compared with metabolomics data from blood plasma. Biostatistical analyses identified discernible metabolic patterns in both biofluids, enabling differentiation of PD patients from healthy controls (OOB error < 1%). Metabolomic breath profiling of PD patients yielded 7 significant metabolites putatively identified as tricosanoic acid, docosanamide, eicosanoic acid, homophytanic acid, nonadecyl-MG, stearic acid, and palmitic acid in PD patients, irrespective of the genetic status. Five of these metabolites were also found in unaffected carriers of pathogenic variants in *LRRK2* when compared to controls. Most of the proposed structures are intermediates in fatty acid metabolism, introducing new candidate biomarkers for breath analysis in PD, although their identities require MS/MS confirmation. Breath analysis effectively distinguishes between PD patients and healthy controls and can identify metabolites that could serve as noninvasive biomarkers for PD, potentially including its presymptomatic stage.

## Introduction

Over the past decade, Parkinson’s disease (PD) has shown the most rapid growth among neurological disorders, affecting more than 10 million individuals worldwide and ranking as the second most prevalent neurodegenerative condition^1,2^. The role of genetic factors in the etiology of PD has also become increasingly clear^3^. Accordingly, approximately up to 15% of PD cases are associated with causative pathogenic variants in *SNCA*, *LRRK2*, *VPS35*, *RAB32*, *CHCHD2*, *PRKN*, *PINK1*, *PARK7* or in *GBA1*, the strongest known risk factor for the development of PD^3–7^.

To date, the timely diagnosis of PD has remained challenging, as clinical criteria primarily assess the extent of decline in motoric function^8^ through clinical signs like bradykinesia, resting tremor, rigidity, and postural instability, which generally only develop once 50-80% of the dopaminergic neurons have already been depleted^9,10^. Accurate diagnostic tests are essential, especially in its early, or even prodromal phase, to enable timely case ascertainment, enrollment into clinical trials and, potentially, tracking of response to disease-modifying therapy. Molecular markers hold promise, as they may reveal pathological changes even before any signs or symptoms occur. Research has therefore focused on identifying such biomarkers in body fluids like blood, saliva, cerebrospinal fluid (CSF)^11–14^, leading to the advent of alpha-synuclein seed amplification assays (aSyn-SAAs) to improve diagnostic accuracy in PD^14–16^, as well as recent efforts to establish the first biological classifications of PD^17,18^. However, the dependence on invasive CSF sampling limits the use of such methods for large-scale screening, resulting in the need for additional non-invasive approaches. Several omics approaches like transcriptomics, proteomics, and metabolomics studies have shown promise in identifying biochemical biomarkers^19–21^ but to date, no specific diagnostic biomarker for PD has yet been validated for use in clinical practice^13,22^. Alternative approaches using sensitive, robust, and clinically applicable analytical techniques may therefore still have a crucial role to play. Recent investigations have reported that individuals with PD emit a distinct ‘musky’ odor that can be identified in the prodromal phase by hyperosmic individuals^23^. Volatile metabolites in sebum, produced by the skin’s sebaceous glands, may contribute to this signature. Other authors have found metabolic changes in sebum samples, suggesting a lipid dysregulation in PD^24^. These intriguing results suggest that the biochemical alterations in this excretory body fluid in PD patients warrant more comprehensive study. Another non-invasively collected yet underexplored excretory biomaterial is breath. Recent reports suggest that the pathological mechanisms of diseases can significantly influence the composition of breath^25^. Breath analysis has shown promise for diagnosing and monitoring various conditions, including Alzheimer’s disease and PD. However, its application in PD research remains in its infancy. Most studies have predominantly focused on volatile organic compounds (VOCs) using complex (sensor) systems^26–29^, while non-volatile organic compounds (nVOCs) in breath have largely been neglected. Investigating nVOCs could both reveal additional biomarkers and allow a more comprehensive understanding of breath and disease-related changes. To this end, we recently demonstrated the potential of analyzing the non-volatile metabolome of ‘healthy’ breath using a simple filter-based device^30^.

In this study, we applied this untargeted metabolomic approach using extreme-resolution Fourier-transform ion cyclotron resonance mass spectrometry (FT-ICR-MS) combined with diverse biostatistical analyses (1) to characterize the non-volatile breath-signature of PD patients with and without pathogenic genetic variants in PD-linked genes (2) to provide a comprehensive overview of the altered metabolomic patterns in the breath of diseased versus healthy individuals and (3) to examine potential metabolomic changes in healthy individuals carrying pathogenic variants. This work establishes the first non-volatile metabolomic breath profile in pathogenic or strong coding risk variant (*LRRK2*, *GBA1*, *PRKN*) PD and idiopathic PD (IPD) patients, presenting significant metabolites that may be indicative of PD.

## Results

The exhaled breath sample group consisted of 73 PD patients (female: 23, male: 50, age range 29–84 years) and 90 healthy participants (female: 37, male: 53, age range 20–60 years) with a BMI range 19.0–25.0 kg m^−2^. To validate the breath analysis findings, metabolite patterns of exhaled breath were compared to those of blood plasma for which a blood plasma sample group was recruited consisting of 91 PD patients (female: 46, male: 45, age range: 29–84) and an independent group of 78 healthy participants (female: 39, male: 39, age range: 25–89) with all participants’ BMI in the 19.0–25.0 kg m^−2^ range. Both patient groups were further categorized into four PD subgroups, including idiopathic PD (no detected pathogenic variant in a known PD gene) and three monogenic PD groups with pathogenic variants in *GBA1*, *LRRK2*, or *PRKN*, respectively. Additionally, 4 and 11 non-manifesting carriers were included in the exhaled breath and blood analysis groups, respectively (Table 1). This investigation analyzed 498 exhaled breath and 180 blood plasma samples, comprising 167 and 180 participants, respectively. Table 2 summarizes the baseline characteristics of PD patients in the exhaled breath group.

**Table 1:** Characteristics of the participants

A: Exhaled breath analysis						
Characteristic	PD^+^				PD^−^	Healthy controls
	*LRRK2*	*GBA1*	*PRKN*	*Idiopathic*	*LRRK2*	No pathogenic variant
Overall *n* = 167	12	35	6	20	4	90
Female	3	11	4	6	1	37
Male	9	24	2	14	3	53
Age [years]	29–84 (mean: 60 ± 10)				27–44 (mean: 35 ± 8)	20–60 (mean: 30 ± 7)

B: Blood plasma analysis							
Characteristic	PD^+^				PD^−^		Healthy controls
	*LRRK2*	*GBA1*	*PRKN*	*Idiopathic*	*LRRK2*	*GBA1*	No pathogenic variant
Overall *n* = 180	13	35	4	39	8	3	78
Female	3	11	2	17	3	1	39
Male	10	24	2	22	5	2	39
Age [years]	35–85 (mean: 61 ± 9)				29–65 (mean: 50 ± 14)		25–89 (mean: 69 ± 15)

Participant groups include individuals diagnosed with Parkinson’s disease (PD+), either with a pathogenic or strong coding risk variant in *LRRK2*, *GBA1*, or *PRKN* or without pathogenic variants (idiopathic PD+). Non-manifesting carriers are healthy unaffected individuals carrying a pathogenic variant in one of these genes (PD–), while healthy controls (HC) are unaffected individuals without any known pathogenic variant.

**Table 2:** Baseline characteristics of PD patients

PD Patients	Overall	*LRRK2*	*GBA1*	*PRKN*	Idiopathic
Disease duration [years]	7 ± 4	8 ± 5	6 ± 4	9 ± 4	7 ± 4
MDS–UPDRS-III score (on)	29 ± 10	24 ± 10	30 ± 9	45 ± 26	29 ± 12
Hoehn and Yahr stage (range)	2 (1–4)	3 (2–3)	2 (1–4)	2 (1–2.5)	2 (1–3)
LEDD [mg]	544 ± 393	712 ± 531	550 ± 353	243 ± 158	532 ± 392

Clinical data of the exhaled breath group, categorized by mutation status. Values are reported as mean ± standard deviation. Disease duration refers to the years since diagnosis. Medications other than levodopa taken by PD patients in this study included opicapone, entacapone, rotigotine, ropinirole, pramipexole, piribedil, rasagiline, safinamide, amantadine, and rivastigmine.

*MDS-UPDRS* Movement Disorder Society – Unified Parkinson’s Disease Rating Scale, Part III (motor section), *LEDD* levodopa equivalent daily dose.

### Core metabolome

**Fig. 1:**
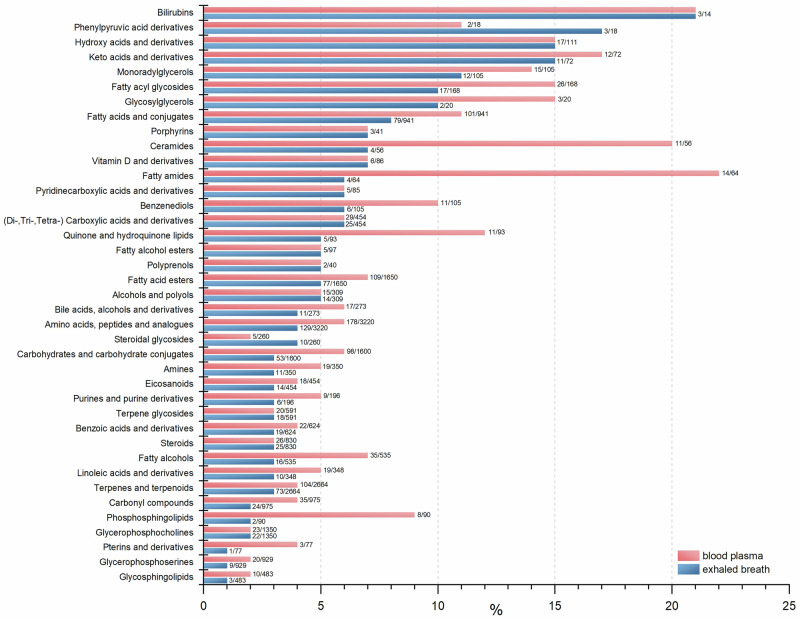
The non-volatile core metabolome of PD patients classified by chemical subclasses. Core Metabolome analyses were performed using MetaboAnalyst 6.0, employing metabolite set enrichment analysis with 1250 sets of chemical sub-class metabolites. All metabolites present solely in samples of healthy participants were removed from the dataset. Out of 2199 detected metabolites, 1954 were utilized for the metabolome analysis of exhaled breath. Additionally, among the 3502 metabolites identified in blood plasma samples, 2898 metabolites were selected for the analysis. Non-naturally occurring metabolites that are according to Human Metabolome Database (HMDB) part of the human exposome were not excluded. The first value displayed at the bars represents the actual number of detected metabolites in both body fluids while the second value denotes the maximum number of potential metabolites registered in the HMDB. The *x*-axis summarizes the resulting fraction of the respective chemical class found in both body fluids in percent (%). Only classes accounting for >1% of the total hits are depicted, ensuring a substantial presence of the classes in the dataset.

In total, 2199 and 3502 chemical formulas and their corresponding postulated metabolites were found in exhaled breath and blood plasma samples, respectively, of all participants. The full sets of annotated metabolites were used to characterize the metabolomic profiles in both body fluids of PD patients. We utilized the Human Metabolome Database (HMDB)^31^ 2023, which provided corresponding HMDB IDs for metabolites. Before analysis, all metabolites present only in healthy participants were removed from the dataset. As a result, 2898 metabolites detected in blood plasma and 1954 metabolites identified in exhaled breath samples were selected for the metabolite set enrichment analysis (MSEA). Since not every HMDB ID is included in MetaboAnalyst 6.0^32^, the MSEA yielded 1087 and 1475 metabolites for exhaled breath and blood plasma, respectively. These metabolites were categorized into chemical subclasses (Fig. 1). The fraction of the respective chemical class was calculated by dividing the number of detected metabolites by the number of the maximum potential hits. The exhaled breath of PD patients revealed a spectrum of metabolite subclasses originating from diverse metabolic pathways akin to those detected in blood plasma. However, both plasma and breath datasets also displayed discernible differences regarding metabolite subclasses such as ceramides, fatty amides, fatty acid esters, amino acids, or carbohydrates, which were identified in exhaled breath at relatively lower levels compared to blood plasma. Figure 1 depicts an overview of the metabolomic profile of PD patients and compares the chemical subclasses found in both body fluids.

### Metabolomic differentiation of PD patients and healthy controls

**Fig. 2:**
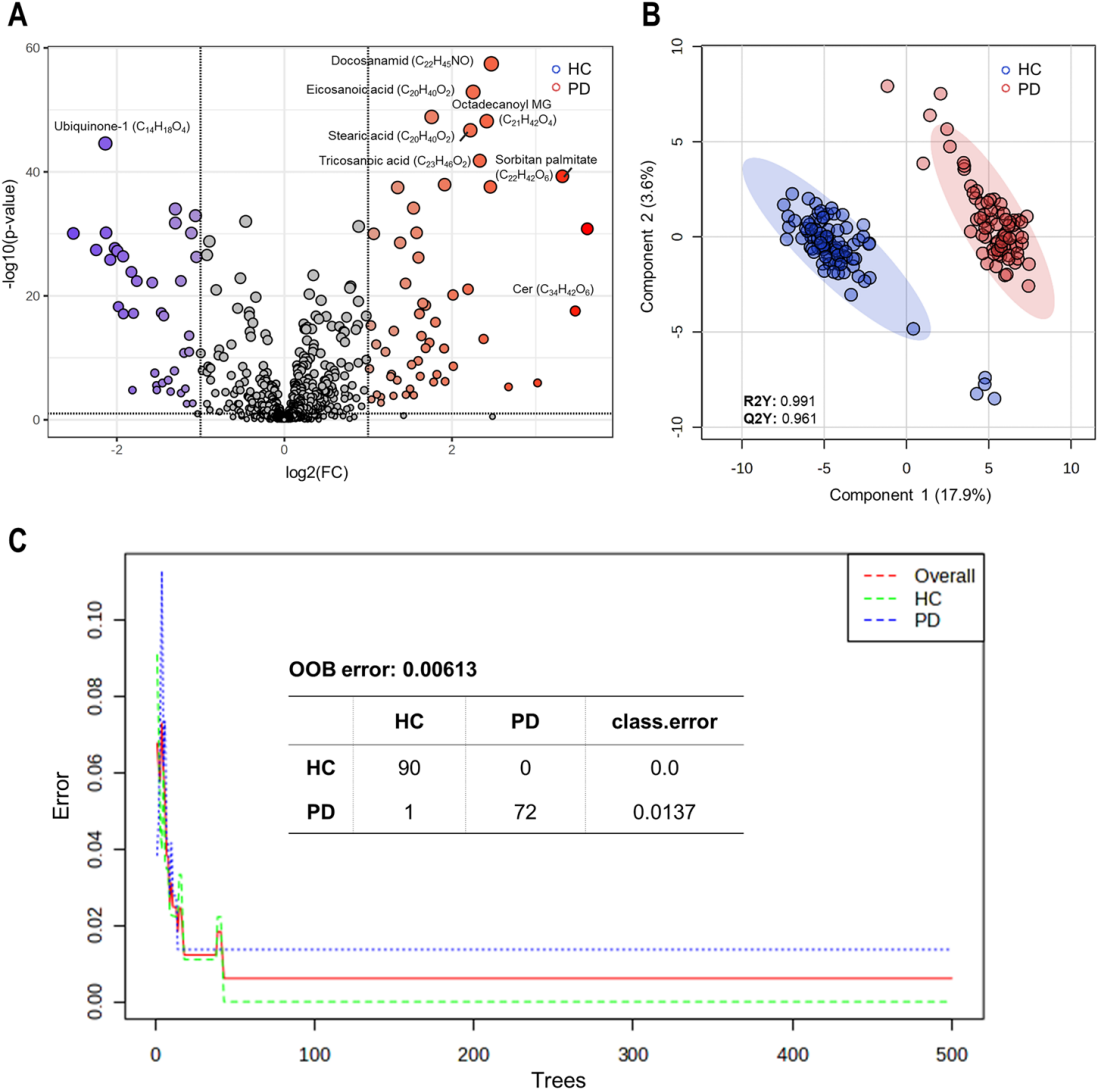
Elaboration of metabolic patterns in exhaled breath using three biostatistical methods. **A** Univariate analysis: 89 metabolites identified by volcano plot differing between PD patients and healthy controls (HC), meeting the criteria of a fold change (FC) threshold > 2.0 and an adjusted *p*-value (FDR correction) of <0.1. Metabolites exhibiting a log2(FC) > 0 (red) indicate elevated intensities in PD patients, while those with a log2(FC) < 0 (blue) signify higher levels in HC. **B** Multivariate analysis: Scores plot of PLS-DA analysis depicting a clear separation of the metabolite profile of PD patients (red) and HC (blue) with R2Y = 0.991 and Q2Y = 0.961 for five components. **C** Classification analysis: Random forest classification with 40 predictors and 500 trees. Based on their metabolite profile, 72 of 73 PD patients as well as all HC were correctly classified, resulting in an out-of-bag (OOB) error of 0.00613. All calculations and analyses were carried out using MetaboAnalyst 6.0.

The datasets were analyzed to identify PD-specific metabolites distinguishing PD patients, with or without pathogenic variants in known PD genes, from healthy controls (HC). The first analysis compared metabolomic patterns of PD patients and HC in exhaled breath and blood plasma using volcano analysis, partial least squares discriminant analysis (PLS-DA), and random forest classification. This approach revealed discernible differences in the metabolomic profiles of PD patients and HC. Figure 2 summarizes the results obtained from all three statistical approaches. The volcano analysis of breath samples revealed 89 significant hits differing between PD patients and HC (FC > 2.0, adjusted *p*-value false discovery rate (FDR) correction) < 0.1) (Fig. 2A). Eighty four of these metabolites remained significant at an FDR-adjusted *p*-value < 0.05, whereas 5 metabolites were no longer significant. Additionally, scores plot from PLS-DA analysis exhibited a clear separation between PD and HC clusters (R2Y = 0.991 and Q2Y = 0.961 for five components) (Fig. 2B). Lastly, employing random forest classification, the algorithm categorized the dataset with a high degree of accuracy (Fig. 2C). All HC were correctly assigned, while 72 of 73 PD patients were categorized as such with a measured out-of-bag (OOB) error rate of <1%.

Employing the same methodology and biostatistical analyses for blood plasma samples yielded comparable results. All three methods distinguished the metabolomic profiles of PD patients from HC (Supplementary Fig. 1). Supplementary Fig. 1A shows the volcano analysis of blood plasma samples, identifying 82 significantly different metabolites between PD patients and HC (FC > 2.0, adjusted *p*-value (FDR correction) < 0.1). Similar to the exhaled breath analysis, the generated PLS-DA scores plot demonstrated a distinct cluster separation between PD and HC (R2Y = 0.984 and Q2Y = 0.921 for five components) (Supplementary Fig. 1B). The random forest algorithm correctly classified all HC and PD patients based on their metabolite profiles, achieving a classification accuracy of 100%. To summarize, all three tests showed discernible differences in metabolic patterns between PD patients and healthy individuals in both body fluids. Next, to cross-validate the biostatistical methods and to pinpoint key metabolites that could discriminate between samples from PD patients and HC, we compared the top 15 metabolites from PLS-DA (VIP score >2.5) and random forest analysis (mean decrease accuracy >0.001) (Supplementary Figs. 2 and 3) in both fluids. None of the top 15 exhaled breath metabolites were detected in blood plasma using PLS-DA (Supplementary Fig. 3A) and random forest (Supplementary Fig. 3B).

**Fig. 3:**
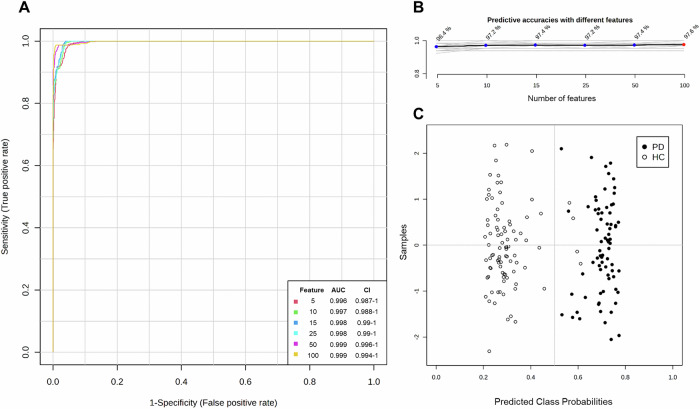
Data-based PD prediction via multivariate exploratory ROC curve analysis of exhaled breath samples. PLS-DA and its built-in feature were selected as the classification method and the ranking method, respectively. The analysis was conducted using two latent variables. **A** ROC curve analyses for *n* = 5, 10, 15, 25, 50, and 100 independent metabolite features (without combining) in PD patients and healthy controls (HC). AUC and 95% confidence intervals (CI) were calculated by Monte Carlo cross-validation (MCCV) using balanced sub-sampling **B** Predictive accuracies (*y*-axis) in percent (%) for *n* = 5, 10, 15, 25, 50, and 100 independent metabolite features. **C** Predicted class probabilities using the best classifier (*n* = 10) based on the AUC. Using this model, all PD patients (*n* = 73) were accurately predicted, while four of 90 healthy individuals were classified as PD patients, currently false positively. Same results were obtained with *n* ≥ 15 features. Since a balanced sub-sampling technique was implemented during model training, the classification boundary consistently aligns with *x* = 0.5, indicated by the dotted line. ROC curve analyses were carried out using MetaboAnalyst Biomarker Analysis 6.0.

**Fig. 4:**
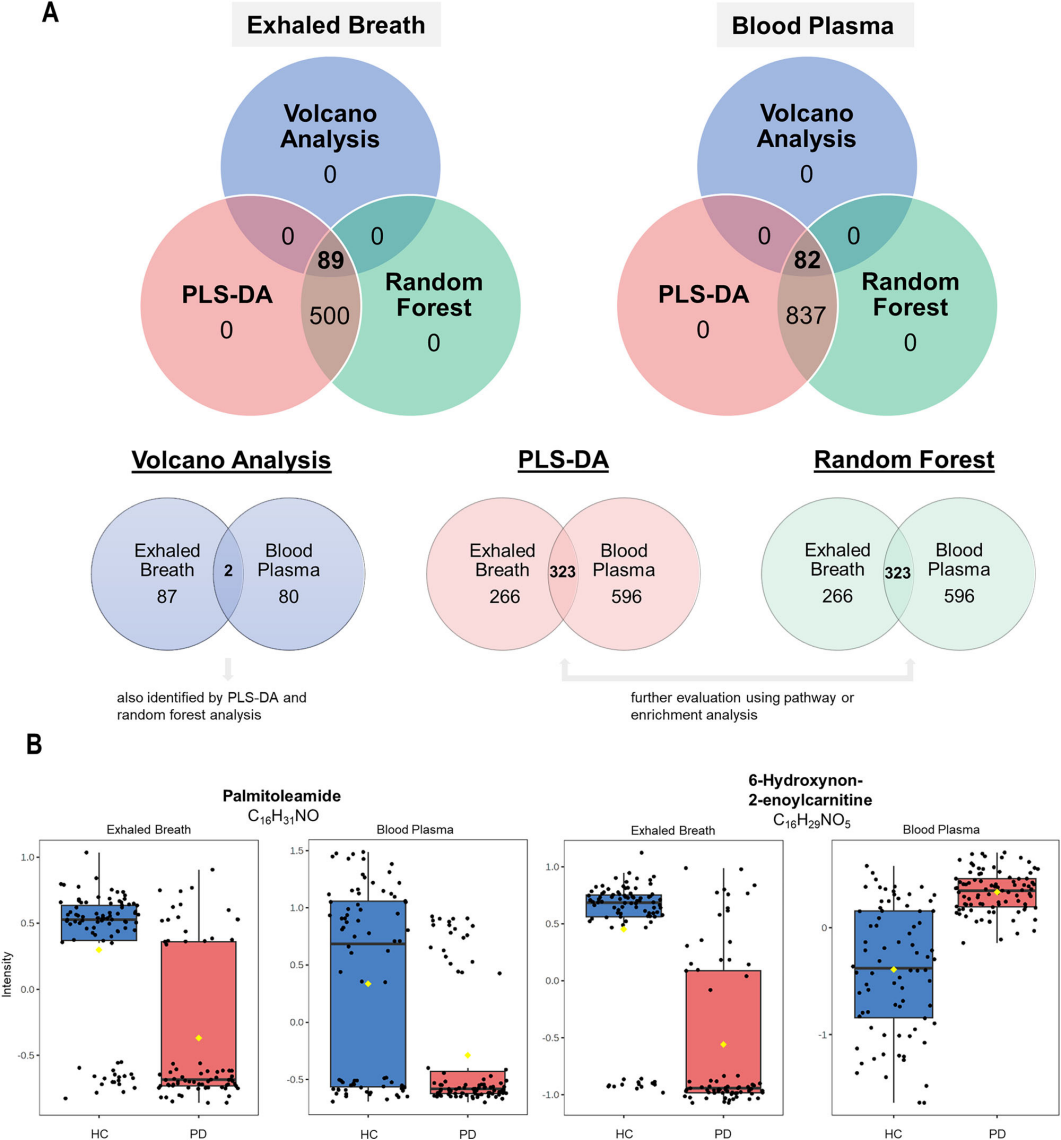
Venn analysis and comparison of significant metabolites identified in all tests. **A** The significant results obtained from all three tests (volcano analysis, PLS-DA, random forest classification) combined in a Venn diagram resulted in 89 and 82 significant metabolites, commonly identified in all three tests in exhaled breath and blood plasma, respectively. **B** A second Venn analysis comparing those aforementioned hit features between exhaled breath and blood plasma reveals two common metabolites significantly different between PD patients and healthy controls (HC) in all tests. 323 common metabolites were detected exclusively in two of the three tests (PLS-DA, random forest) in both body fluids. **C** Box plots of the two significant metabolites with chemical formula and putative identity found across all biostatistical approaches in both exhaled breath (left) and blood plasma (right), respectively. The results were significant between PD patients (red, *n* = 73 biologically independent exhaled breath and *n* = 91 biologically independent plasma samples) and healthy controls (HC) (blue, *n* = 90 biologically independent exhaled breath and *n* = 78 biologically independent plasma samples). Black dots represent the values from all samples. The box and whiskers summarize the normalized values, with the center line presenting the median. The mean value is indicated as a yellow diamond.

To further evaluate the data-driven PD prediction, we conducted an additional multivariate exploratory ROC curve analysis for both exhaled breath (Fig. 3) and blood plasma (Supplementary Fig. 4). Figure 3A and Supplementary Fig. 4A illustrate the ROC curve analyses for independent metabolite features (*n* = 5, 10, 15, 25, 50, and 100). Comparing the ROC curves, both models classified PD patients and HC with AUCs of 0.99–1 (CI 95%). Using this approach, both datasets predicted PD accurately (>96%) when considering only 10 metabolite features (Fig. 3B and Supplementary Fig. 4B). While adding more features showed enhanced prediction accuracy, the results indicate that up to 25 features were already sufficient for a reliable prediction of PD in both body fluids. In breath analysis, all 73 PD patients were accurately predicted solely based on a metabolomic pattern utilizing only 10 features (Fig. 3C). Similarly, when evaluating blood plasma, all HC were correctly identified, while only 5 out of 91 PD patients were misclassified. This result was achieved using a set of 25 features selected as the best classifier model according to the AUC (Supplementary Fig. 4C). Comparing these results with those obtained from random forest classification (Fig. 2C and Supplementary Fig. 1C), both statistical approaches accurately distinguished PD patients from HC. After validating the results obtained by biostatistical methods, the key metabolites responsible for this differentiation were examined. To explore features identified by multiple statistical methods across both body fluids, we conducted a Venn analysis, combining all significant metabolites found in volcano analysis, PLS-DA and random forest analysis (Fig. 4). This investigation yielded 89 metabolites in exhaled breath and 82 in blood plasma that differed between PD and HC and were identified across all three statistical tests (Fig. 4A). No metabolites were exclusive to any single test. Rather, 500 and 837 metabolites were commonly identified between the PLS-DA and random forest analyses for exhaled breath and blood plasma, respectively. To explore potential ‘between fluid’ overlaps, a subsequent Venn analysis was conducted. This analysis compared the results from the three statistical tests for both fluids (Fig. 4B) to identify shared biomarkers between breath and blood plasma that could contribute to a unified PD-specific metabolomic profile. This process consistently identified 323 significant metabolites using PLS-DA and random forest for both body fluids. However, a substantial number of the metabolites detected in body fluids were found to be unique to a particular matrix, with 266 exclusive to exhaled breath and 596 to blood plasma. Only palmitoleamide (C_16_H_31_NO) and 6-hydroxynon-2-enoylcarnitine (C_16_H_29_NO_5_) were commonly detected across all statistical tests in both exhaled breath and blood plasma (Fig. 4C) but were found to differ in distribution between body fluids: while palmitoleamide (C_16_H_31_NO) exhibited consistent results, 6-hydroxynon-2-enoylcarnitine (C_16_H_29_NO_5_) showed higher intensity levels in PD blood plasma but lower in exhaled breath samples when comparing patients to controls. From the Venn analysis, we selected the 15 most consistent metabolites (*p* value « 0.001, VIP scores >1.5, mean decrease accuracy >0.0001) and calculated the expression ratios between both groups. Tables 3 and 4 outline these metabolites for exhaled breath and blood plasma, respectively. Although the top 15 metabolites identified in breath and blood plasma showed no overlap, several were still present in the broader set of shared metabolites (*n* = 323). Specifically, 7 of the top 15 breath metabolites (docosanamide, nonadecanoic acid, tricosanoic acid, sorbitan palmitate, 2-hydroxy-3-methyl-pentanoic acid, 2-methyl-3-hydroxy-butyric acid, and ubiquinone-1) were found among the 323 metabolites common to both specimen types (Fig. 4B and Table 3). Likewise, three of the top 15 plasma metabolites (C_9_H_7_NO, C_14_H_22_N_2_O, and 3-methoxytyrosine) were also included in this shared metabolite set (Supplementary Table 1).

**Table 3:** The 15 most relevant metabolites in exhaled breath of PD patients obtained from Venn analysis

Chemical formula	Putative identity	Expression ratio 1/FC (95% CI)	*t*-test (adj. *p*-value)	PLS-DA (VIP score)	RF (MDA)
C_22_H_45_NO	Docosanamide^#^	↑ 0.18 (0.16–0.20)	4.45E-62	3.16	0.015
C_20_H_40_O_2_	Eicosanoic acid	↑ 0.21 (0.19–0.24)	5.46E-57	2.98	0.023
C_19_H_38_O_2_	Nonadecanoic acid^#^	↑ 0.30 (0.27–0.33)	7.59E-53	2.56	0.024
C_21_H_42_O_4_	Octadecanoyl MG	↑ 0.19 (0.16–0.22)	5.49E-52	3.22	0.025
C_18_H_36_O_2_	Stearic acid	↑ 0.22 (0.19–0.25)	3.13E-50	2.99	0.020
C_23_H_46_O_2_	Tricosanoic acid^#^	↑ 0.20 (0.17–0.23)	3.77E-45	3.14	0.029
C_22_H_42_O_6_	Sorbitan palmitate^#^	↑ 0.10 (0.08–0.13)	4.49E-42	3.92	0.011
C_22_H_44_O_4_	Nonadecanoyl MG	↑ 0.27 (0.23–0.31)	3.14E-41	2.68	0.028
C_16_H_32_O_2_	Palmitic acid	↑ 0.40 (0.36–0.44)	5.37E-41	2.15	0.007
C_24_H_48_O_2_	Tetracosanoic acid	↑ 0.18 (0.15–0.22)	1.41E-40	3.23	0.029
C_19_H_38_O_4_	Hexadecanoyl MG	↑ 0.35 (0.31–0.40)	1.10E-37	2.32	0.006
C_6_H_12_O_3_	2-Hydroxy-3-methyl-valeric acid^#^	↓ 2.48 (2.23–2.76)	6.19E-37	2.20	0.031
C_5_H_10_O_3_	2-Methyl-3-hydroxy-butyric acid^#^	↓ 2.48 (2.21–2.78)	1.46E-34	2.07	0.016
C_17_H_34_O_2_	Heptadecanoic acid	↑ 0.48 (0.44–0.53)	4.08E-33	1.84	0.003
C_14_H_18_O_4_	Ubiquinone-1^#^	↓ 4.43 (3.64–5.39)	3.75E-32	2.70	0.002

The metabolites were identified in all three biostatistical methods with expression ratios obtained from a fold change analysis (FC = 2.0) with 95% CI, adjusted *p*-values « 0.001 (*t*-test, volcano analysis), VIP scores >1.5 (PLS-DA) and mean decrease accuracy values > 0.0001 (random forest, RF), with arrows indicating either higher or lower levels in PD patients compared to healthy controls. Metabolites labeled with a hash mark (^#^) were identified in Venn analysis of blood plasma as well. Nonadecanoic acid, tetracosanoic acid, and 2-hydroxy-3-methylvaleric acid were no longer significant when adjusting for age and/or gender.

*MG* monogylceride.

**Table 4:** Top metabolites in breath of unaffected participants carrying a *LRRK2* mutation

Chemical formula	Putative identity	Expression ratio 1/FC (95% CI)	*t*-test (adj. *p*-value)
C_21_H_42_O_4_	Octadecanoyl MG	↑ 0.11 (0.04–0.31)	5,21E-05
C_18_H_36_O_2_	Stearic acid*	↑ 0.15 (0.09–0.24)	1,61E-13
C_20_H_40_O_2_	Eicosanoic acid*	↑ 0.19 (0.08-0.47)	3,51E-04
C_19_H_38_O_4_	Hexadecanoyl MG	↑ 0.19 (0.09–0.38)	4,91E-06
C_19_H_38_O_2_	Nonadecanoic acid	↑ 0.23 (0.12–0.45)	2,21E-05
C_22_H_44_O_4_	Nonadecanoyl MG*	↑ 0.19 (0.08–0.44)	1,66E-04
C_26_H_38_O_8_	1-Methylene-5alpha-androstan-3alpha-ol-17-one glucuronide	↑ 0.18 (0.09–0.34)	5,81E-07
C_16_H_32_O_2_	Palmitic acid*	↑ 0.27 (0.16–0.44)	5,61E-07
C_21_H_42_O_2_	Homophytanic acid*	↑ 0.32 (0.20–0.50)	1,98E-06
C_17_H_34_O_2_	Heptadecanoic acid	↑ 0.29 (0.15-0.56)	2,36E-04
C_25_H_39_O_11_P	Phosphatidic acid (PA)	↑ 0.39 (0.28–0.54)	1,03E-07
C_6_H_12_O_3_	2-hydroxy-3-methylvaleric acid (HMVA)	↓ 2.56 (1.61–4.08)	9,91E-05
C_5_H_10_O_3_	3-hydroxy-2-methylbutanoic acid	↓ 2.17 (1.70–2.78)	4,69E-09

Metabolites were identified across various biostatistical tests (PLS-DA, volcano plot, random forest), as well as in biomarker analysis using univariate ROC curve analyses. Only metabolites meeting the criteria of a VIP Score >1.5, *p*-value < 0.001, mean decrease accuracy >0.0001, and a ROC AUC > 0.98 were selected. The metabolites were further analyzed based on their expression ratios obtained from a fold change analysis (FC = 2). Arrows indicate either higher or lower levels in healthy participants with *LRRK2* mutation compared to healthy participants without mutation. Metabolites labeled with an asterisk (*) were also identified and characterized among the top 7 metabolites differing between PD patients and healthy controls with a similar distribution. Nonadecanoic acid and HMVA were found no longer significant when adjusting for age and/or gender.

*MG* monogylceride.

**Fig. 5:**
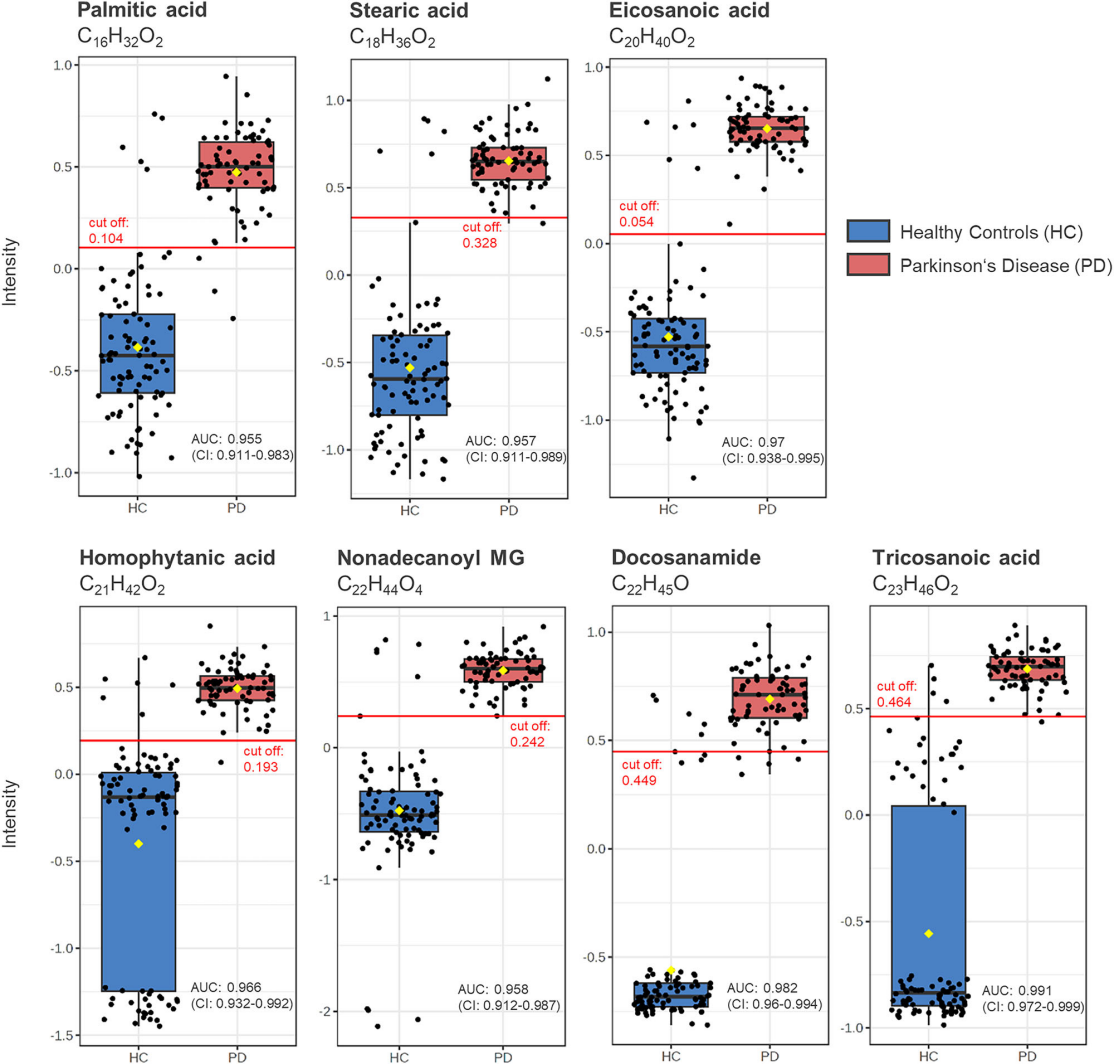
The top 7 significant metabolites identified in exhaled breath using univariate ROC curve analyses. The univariate ROC analysis used 500 bootstrappings for calculating the 95% confidence interval (CI). Only those metabolites were selected that were identified in Venn analysis across volcano analysis (FC > 2.0, adjusted *p*-value (FDR correction) <0.1), PLS-DA (VIP score > 2.5) and random forest analysis (mean decrease accuracy >0.001). The box plots depict significant differences between the group of PD patients (red, *n* = 73 biologically independent samples) and healthy controls (HC) (blue, *n* = 90 biologically independent samples). Black dots represent the values from all samples. The box and whiskers summarize the normalized values with the center line presenting the median. The mean value is indicated as a yellow diamond. The red line denotes the optimal cut-off values (closest to the top-left corner) obtained from univariate ROC analysis. MG monoglyceride.

In order to identify not only the most statistically robust but also the most relevant metabolites as potential biomarker candidates, the analysis proceeded with a univariate classical ROC analysis of the top 10 metabolites with an AUC > 0.96. Age- and gender-adjusted analyses confirmed the robustness of the findings, with 7 of 10 metabolites remaining statistically significant while nonadecanoic acid, 2-hydroxy-3-methylvaleric acid (HMVA), and tetracosanoic acid were no longer significant. Figure 5 exhibits boxplots of the top 7 significant breath metabolites identified through all statistical analyses. Five of the 7 metabolites, including tetracosanoic acid, tricosanoic acid, eicosanoic acid, homophytanic acid, stearic acid, and palmitic acid, were elevated in PD patients. To ascertain the significance of these metabolites in PD patients, irrespective of pathogenic variants, we performed a one-way ANOVA with Tukey HSD correction (*p*-value < 0.05,) which showed that all these metabolites exhibit significantly higher levels across all subgroups compared to HC.

None of these metabolites were found among the 82 significant metabolites of blood plasma identified through Venn analysis (Fig. 4A). However, tricosanoic acid, HMVA, nonadecanoic acid and homophytanic acid were found among the 323 shared metabolites between exhaled breath and blood plasma. Re-evaluating the top 15 metabolites of blood plasma (Supplementary Table 1) with an additional univariate classical ROC analysis, only 4 of these metabolites (C_12_H_26_O, C_2_H_7_O_3_P, C_14_H_22_N_2_O, C_12_H_24_O) exhibited an AUC greater than 0.96.

The above mentioned statistical analyses revealed significant differences between PD and HC groups. However, the PD group was not stratified by mutation status in these aforementioned analyses. To assess the impact of pathogenic variants on the observed metabolic profile, PD patients were divided into two groups: idiopathic PD (IPD) and PD with mutation (mPD). Statistical tests were conducted for both groups compared to HC. The results were consistent, with identical distributions when comparing the top 20 outcomes from volcano plots, PLS-DA, and random forest analyses, similar to the overall PD and HC comparison.

### Analysis of metabolomic patterns in PD patients with and without genetic variants

Metabolomic patterns in PD patients with (mPD) and without (IPD) genetic variants in three known PD genes (*GBA1*, *LRRK2*, and *PRKN)* were measured and subject to between-group statistical analyses (Supplementary Information). The volcano analysis identified 10 and 23 significant metabolites in the breath and blood plasma samples, respectively of the IPD and mPD group (Supplementary Figs. 5 and 6). These particular metabolites were not identified as significantly different when comparing all PD patients to HC. When selecting only naturally occurring metabolites, the volcano plot highlighted N-decanoylglycine (C_12_H_23_NO_3_) and 2-octenoylcarnitine (C_15_H_27_NO_4_) as significantly elevated in breath of the mPD group compared to IPD patients (Supplementary Fig. 5).

Further multivariate analysis and classification of all PD subgroups were conducted. Supplementary Fig. 7 summarizes the PLS-DA and random forest classification outcomes. PLS-DA revealed a clear separation of PD patients with and without pathogenic variants in both body fluids (Supplementary Fig. 7A). Additionally, the random forest accurately classified all IPD patients in blood plasma and exhaled breath, with only one exception, while misclassifying 3 of 53 PD mutation carriers in breath and 1 of 51 in blood. Assessing all PD subgroups (IPD, PD_ *GBA1*, PD_*LRRK2*, and PD_*PRKN*), the scores plot separated clusters more effectively in blood plasma compared to breath (Supplementary Fig. 7B). The random forest analysis effectively classified IPD patients and PD patients carrying a *GBA1* mutation in both fluids but showed lower accuracy for *LRRK2* and *PRKN* subgroups, increasing OOB errors in both exhaled breath (0.16) and blood plasma (0.09) (Supplementary Fig. 7B). Given the small sample sizes of the genetic subgroups (*GBA1*, *LRRK2* and *PRKN)*, these analyses are underpowered and only exploratory.

### Evaluation of healthy individuals with a pathogenic variant (non-manifesting *LRRK2* mutation carriers)

**Fig. 6:**
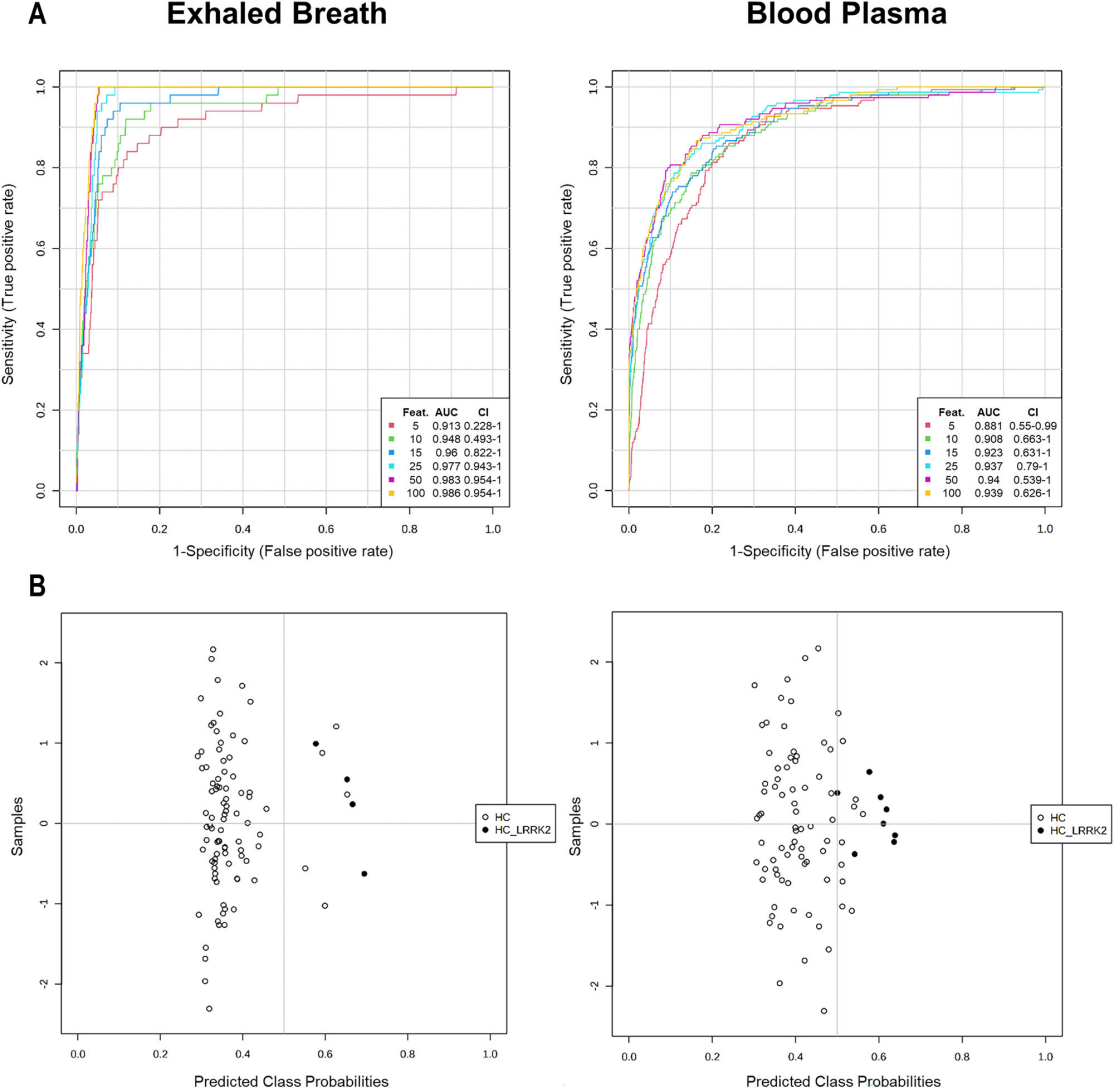
Multivariate exploratory ROC curve analyses of exhaled breath vs. blood plasma for classifying healthy controls with and without *LRRK2* mutation. PLS-DA and its built-in feature was selected for the classification method and the ranking method, respectively. The analysis was conducted using two latent variables. **A** Comparison of ROC curve analyses of exhaled breath and blood plasma for *n* = 5, 10, 15, 25, 50 and 100 independent metabolite features in healthy controls and healthy individuals with *LRRK2* mutation. AUC and 95% confidence intervals (CI) were calculated by Monte Carlo cross-validation (MCCV) using balanced sub-sampling. **B** Predicted class probabilities using the best classifier (exhaled breath: *n* = 10, blood plasma: *n* = 15) based on the AUC. Using this model, all 4 and 8 healthy controls carrying an *LRRK2* mutation were accurately predicted and differed from healthy controls by metabolite patterns in exhaled breath and blood plasma, respectively. Since a balanced sub-sampling technique was implemented during model training, the classification boundary consistently aligns with *x* = 0.5, indicated by the dotted line. ROC curve analyses were carried out using MetaboAnalyst Biomarker Analysis 6.0.

Subsequent analysis focused on the small group of 4 healthy individuals harboring a pathogenic *LRRK2* variant. Due to the sample size, these results are also exploratory. Initially, a multivariate ROC curve analysis was employed for classification (Fig. 6). The exhaled breath analysis exhibited higher ROC AUC values compared to blood plasma, with respective values of 0.98 and 0.94 for 25 features (Fig. 6A). This analysis discriminated the 4 unaffected individuals carrying a pathogenic variant in *LRRK2* from other HC. When examining the predicted class probabilities, 5 of 90 healthy individuals were misclassified as mutation carriers in exhaled breath, while 10 of 78 were misclassified in the blood plasma group (Fig. 6B). However, this model accurately identified all HC individuals with an *LRRK2* variant (exhaled breath: *n* = 4 and blood plasma: *n* = 8).

Next, PLS-DA, volcano plot, random forest and a classical univariate ROC curve analysis were conducted to identify metabolites that differed significantly between the group of non-manifesting *LRRK2* mutation carriers and HC. Table 4 presents the top metabolites detected in exhaled breath when applying the aforementioned tests (*p*-value < 0.001, VIP score > 1.5, mean decrease accuracy >0.0001, and ROC AUC > 0.98). The metabolites presented in Table 4 were compared to those found in the comparison between PD and HC. Notably, 5 out of the top 7 metabolites identified as different between PD and HC (Fig. 5) were also significantly different in non-manifesting *LRRK2* carriers compared to HC. These remained statistically significant after adjusting for age and gender.

**Fig. 7:**
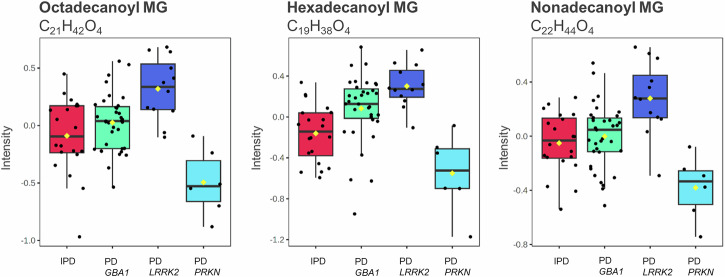
Box plots of three significant metabolites identified in the exhaled breath of PD subgroups. Significant findings obtained from a one-way ANOVA combined with a Tukey honestly significant difference (HSD) test for FDR correction (*p*-value < 0.05). Differences were observed between all PD subgroups (1) IPD patients (red, *n* = 20 biologically independent exhaled breath samples), (2) PD_ *GBA1* (green, *n* = 35 biologically independent exhaled breath samples), (3) PD_*LRRK2* (blue, *n* = 12 biologically independent exhaled breath samples) and (4) PD_*PRKN* (turquoise, *n* = 6 biologically independent exhaled breath samples). The PD patients with a *LRRK2* mutation showed elevated levels compared to the other PD subgroups. All three metabolites showed elevated levels in PD patients compared to healthy controls. Black dots represent the values from all samples. The box and whiskers summarize the normalized values with the center line presenting the median. The mean value is indicated as a yellow diamond. These three metabolites were also found to be significant in a univariate ROC curve analysis comparing healthy controls to healthy individuals carrying a *LRRK2* mutation (see Table 4). MG monoglyceride.

Moreover, non-manifesting *LRRK2* carriers exhibited elevated monoglycerides (MG) levels (Table 4). A one-way ANOVA of MG levels across PD subgroups revealed that *LRRK2*-PD patients showed significantly higher levels compared to other PD subgroups (Fig. 7). MG levels were also found to be significantly higher in PD patients compared to HC, irrespective of the pathogenic variants. The same analysis using blood plasma from non-manifesting *LRRK2* carriers revealed that none of the top 20 blood plasma metabolites in this group matched those that were significantly different between PD patients and HC (Supplementary Table 1). However, the volcano analysis identified 117 metabolites differing between non-manifesting carriers and HC (*p* < 0.001), while PLS-DA and random forest classification detected 317 and 242 metabolites, respectively (VIP score >1.5, mean decrease accuracy >0.0001).

Among the top 20 differentiating metabolites between PD and HC, only octadecanamide (C_18_H_37_NO) achieved a high accuracy via univariate ROC curve analysis (ROC AUC 0.94) comparing non-manifesting *LRRK2* carriers and HC, showing 2.45-fold higher levels in PD than in HC. However, this metabolite did not reach significance specifically for PD patients with an *LRRK2* mutation (one-way ANOVA), but did demonstrate significantly higher levels in the HC group compared to all PD patients.

## Discussion

This breathomics study applied a non-targeted metabolomic approach focusing on the non-volatile organic compounds in breath, as previously tested in healthy individuals^30^. Utilizing ultra-high-resolution FT-ICR-MS facilitated an extremely high sensitivity for distinguishing traces in the femtomole range. Here, we describe the PD “breathome”—the repertoire of breath-derived non-volatile metabolites that consistently and reproducibly differ between PD patients, with and without known pathogenic genetic variants, and HC. This discussion mainly focuses on exhaled breath analysis while using the commonly employed blood plasma as a reference to compare the body fluids.

The pre-processed datasets successfully yielded 2199 and 3502 chemical compounds and their corresponding inferred metabolites in exhaled breath and blood plasma of all participants, respectively. When assigning identities to metabolites, it is important to consider the large number of isobaric compounds sharing the same chemical formula. If multiple potential compounds were attributed, we selected the first listed metabolite from the annotation list. However, for all proposed significant metabolites, plausible alternatives within the annotation list were also explored. Such feasible alternatives will be discussed below. In general, blood plasma yielded over 1000 more metabolites compared to breath. However, it should be considered that the 2199 metabolites detected in breath selectively represent only its non-volatile fraction. Hence, a breathomics approach analyzing both non-volatile and volatile organic compounds might identify even more metabolites. Applying an MSEA enabled the characterization of the core metabolome, identifying 1087 metabolites for exhaled breath and 1475 for blood plasma. These metabolites were successfully categorized into chemical subclasses (Fig. 1), highlighting biochemically versatile molecules with various functions in the human body. Blood plasma appears to be richer in certain chemical subclasses, revealing a greater number of metabolites from specific subclasses, such as but not limited to ceramides, fatty amides, fatty acid esters, amino acids, and peptides, as well as carbohydrates. Notably, the number of carbohydrates and ceramides is nearly double in blood plasma compared to exhaled breath. Despite those differences, other subclasses such as fatty alcohol esters, steroids, glycerophosphocholines, hydroxyl acids, and vitamin D and its derivatives evidently exhibit similar proportions in both compartments. Comparing exhaled breath and blood plasma analyses suggests that breath is equally suited to metabolomic analysis as blood plasma. The substantive similarities between their core metabolome composition indicate that breath analysis can maintain accuracy without significant loss of information. Remarkably, non-invasive breath sampling enables the identification of similar chemical classes from just 30 exhalations. The presented chemical subclass groups in Fig. 1 encompass all metabolites found in two body fluids of PD patients, with the presented metabolites not necessarily depicting the significant differences compared to HC. These differences become more apparent in statistical analyses, which are necessary to identify any distinctive signatures in PD patients.

The ability of exhaled breath analysis to effectively differentiate PD patients from HC, similar to blood plasma metabolomic profiling, was striking and was independent of the biostatistical test applied (Figs. 2, 3 and Supplementary Figs. 1, 4). To assess the robustness of the volcano analysis, we additionally evaluated which metabolites remained significant at an FDR-adjusted *p*-value < 0.05. The large majority (84 out of 89) met this threshold, supporting the stability of the observed metabolic differences despite the use of a more permissive FDR-adjusted *p* < 0.1 cutoff appropriate for early-stage discovery.

These findings, and the corresponding Venn analysis, highlight the potential of exhaled breath as a non-invasive alternative biomaterial for metabolomics studies and potential diagnostic applications. While there is a significant overlap of metabolites found in both biospecimens (*n* = 323), a considerable portion is unique to each. Specifically, 266 metabolites were detected exclusively in exhaled breath and 596 solely in blood plasma (Fig. 4B). The limited overlap between discriminatory metabolites in plasma and breath likely results from fundamental biological and matrix-related differences. Plasma circulates in contact with vascular endothelial cells and primarily reflects systemic metabolism. In contrast, exhaled breath originates from the airway epithelial cell layer^30^ and contains metabolites that either diffuse across the alveolar-capillary barrier from systemic sources or are produced locally by airway epithelial cells, alveolar macrophages, and other immune cells. Additional contributions from the constituents of the airway lining fluid that become aerosolized during exhalation^33^ can introduce compounds not typically present in plasma. Together, these differences in cellular barriers, metabolic origin, and sample matrix in general may provide a biologically plausible explanation for the divergence between plasma and breath metabolite profiles.

When examining the significant hits shared between both fluids, two metabolites consistently emerged as significant across all statistical methods, distinguishing between healthy and diseased individuals (Venn analysis, Fig. 4B). One of the two metabolites is palmitoleamide (C_16_H_31_NO), an amide of palmitoleic acid (monounsaturated fatty acid, 16:1n-7). This metabolite exhibited significantly lower levels in PD patients in both exhaled breath and blood plasma samples. It was first discovered as one of five endogenous primary fatty acid amides (PFAM) in luteal phase plasma with their physiological significance being unclear^34^. However, reports suggest that PFAM play a significant role as cell signaling lipids in the mammalian nervous system^35,36^. Since the biological active oleamide, the most studied PFAM, has been reported as a natural component of CSF^36^, it is likely that other PFAM are also present in CSF, each with distinct roles as signaling lipids. It is conceivable that the concentration and composition of PFAM may change in PD, leading to alterations in CSF compared to that of healthy individuals. Furthermore, the second significant metabolite found in both belongs to the class of acylcarnitines. 6-hydroxynon-2-enoylcarnitine (C_16_H_29_NO_5_) was found to be elevated relative to controls in the plasma of PD patients, but was conversely lower in exhaled breath (Fig. 4C). Interestingly, decreased levels of long-chain acylcarnitines have previously been discussed as potential biomarkers for PD^37^. Assessing the top 15 significant hits identified in the respective fluid via Venn analysis revealed that none of the top metabolites were congruent (Table 3, and Supplementary Table 1). Despite a similar core metabolome composition, the most significant metabolites from various chemical classes appear to differ between biospecimens, suggesting notable differences between the two biomaterials.

An in-depth biostatical analysis of exhaled breath identified the top 7 metabolites that differentiate between PD patients and healthy individuals (Fig. 5). These metabolites were not found among the top hits in blood plasma. Cross-validated through multiple statistical tests, they appear to be the most robust and relevant markers for distinguishing the metabolomic profiles of the two groups. Interestingly, most are linked directly or indirectly to lipid metabolism. Docosanamide (C_22_H_45_O), another fatty acid amide, was found to be more abundant in PD patients compared to HC. While this metabolite has not been previously linked to PD, as a member of the primary fatty acid amide family^38^, it might serve as signaling lipid in the nervous system as well^35,36^. Furthermore, the glycerolipid nonadecanoyl monoglyceride (MG) (C_22_H_44_O_4_) was also elevated in PD patients. The other 5 metabolites, palmitic acid (C_16_H_32_O_2_), stearic acid (C_18_H_36_O_2_), eicosanoic acid (C_20_H_40_O_2_), homophytanic acid (C_21_H_42_O_2_), tricosanoic acid (C_23_H_46_O_2_), belong to the fatty acid family. A pathway analysis using MetaboAnalyst 6.0^32^ revealed that palmitic acid (KEGG: C00249), stearic acid (KEGG: C01530) and eicosanoic acid (KEGG: C06425) play roles in the biosynthesis of unsaturated fatty acids (Supplementary Fig. 8), while stearic acid (C_18_H_36_O_2_) is also involved in mitochondrial β-oxidation. Notably, all of the 7 metabolites showed elevated levels in PD patients, indicating consistent alterations in lipid metabolism, particularly concerning fatty acids. This consistent directionality strengthens the evidence for upregulated lipid metabolic processes in PD. Since some fatty acids like palmitic acid are part of mitochondrial fatty acid synthesis as well, these findings may be a manifestation of the mitochondrial metabolic changes and dysfunction already known to be associated with PD^39–41^. Recent research has also suggested that PD is associated with lipid dysregulation or changes in lipid metabolism^24,42,43^, which fits well with our findings. Levodopa has been discussed to influence brain fatty acid composition, notably increasing arachidonic acid levels and altering the n-3:n-6 polyunsaturated fatty acid ratio^44^. However, the key fatty acid metabolites highlighted in this paper have not yet been directly linked to levodopa treatment. Other potential explanations for the observed lipid dysregulation include differences in nutritional intake, variations in microbiome composition, and altered metabolic profiles due to underlying disorders.

Furthermore, dopaminergic drugs used in PD treatment can also contribute to substantial metabolomic differences, particularly in blood plasma^45^. Since all PD patients were receiving medication, particularly dopaminergic therapies (Table 2), this study evaluated potential pharmacological effects. For instance, 3-methoxytyrosine, likely originating from levodopa, was significantly elevated in the top 15 hits in plasma of PD patients (Supplementary Table 1). As a result, drug-induced metabolites or metabolite patterns may not only cause a misinterpretation in the effective differentiation of both groups but also complicate the identification of endogenous metabolites of interest, as their signals may overlap. Notably, the exhaled breath results did not appear to be associated with the dopaminergic medication, as non-manifesting *LRRK2* carriers still exhibited several metabolites that were found to be significantly altered in the PD group (Fig. 5 and Table 4), despite not receiving dopaminergic medication. Furthermore, all identified metabolites were cross-checked against recent literature on the impact of dopaminergic medication in metabolomics^45^, and none of the top metabolites have been reported to be associated with levodopa or any of the other dopaminergic therapies used by the PD patients included in this study (Table 2). Nevertheless, it remains essential in metabolomics studies to stratify patients by treatment status to better characterize the breathome in PD.

While examining plausible alternative isomers for all proposed significant metabolite identities within the annotation list, one metabolite was identified as particularly noteworthy. According to HMDB^31^, the chemical formula C_20_H_40_O_2_ annotated as eicosanoic acid, can be identified as phytanic acid as well. This branched-chain fatty acid has been linked to adult Refsum disease, an autosomal recessive neurological disorder characterized by symptoms like peripheral polyneuropathy, cerebellar ataxia, anosmia, and hearing loss^46,47^. Hence, chronically elevated phytanic acid levels have been reported to be neurotoxic, as they can initiate astrocyte and neural cell death by activating the mitochondrial pathway of apoptosis^48^. Consequently, there is a possibility that phytanic acid could similarly contribute to neuronal cell damage in PD.

It is also notable that the metabolite 2-hydroxy-3-methylvaleric acid (HMVA) (C_6_H_12_O_3_) generated by l-isoleucine metabolism showed lower intensities in PD patients. This compound has been documented to occur at higher levels in the blood and urine of patients suffering from maple syrup urine disease (MSUD)^49^, an inherited metabolic disease predominantly characterized by neurological dysfunction^50^. Branched-chain amino acid alterations have been linked to neurodegenerative movement disorders like PD^51–54^, however, there is currently no established association between HMVA and PD. Nevertheless, as age- and sex-adjusted analyses suggest that the observed difference in HMVA levels may be influenced by demographic factors, any potential association with PD should be interpreted cautiously.

All the aforementioned results suggest that PD extends beyond being solely a neurodegenerative disease and could be considered a multisystem disorder, affecting various metabolic pathways such as those involving amino acid and fatty acids.

This work not only evaluated potential biomarker candidates for an established diagnosis but also examined metabolic signatures that may indicate presymptomatic pathology in apparently healthy individuals. Multivariate ROC curve analyses accurately differentiated the four healthy individuals carrying a pathogenic variant in *LRRK2* gene from HC based on their metabolomic profiles (Fig. 6). In blood plasma, univariate ROC analysis yielded only one common metabolite, the primary fatty acid amide octadecanamide (C_18_H_37_NO), which was significantly lower in both non-manifesting *LRRK2* carriers and PD patients compared to HC (Supplementary Table 1). Interestingly, one-way ANOVA showed that these decreased levels were not exclusive to PD patients carrying the *LRRK2* mutation, suggesting that this metabolomic signature may reflect a downstream process not unique to *LRRK2*-related pathobiology. Nevertheless, as octadecenamide (C_18_H_37_NO) is the amide of stearic acid, this finding supports the idea that PFAM may play a role in PD. The univariate ROC analysis of exhaled breath revealed interesting results in non-manifesting *LRRK2* carriers, in whom 5 of the 7 top metabolites differentiating PD patients from HC (Fig. 5) were also found to be significantly different (Table 4) and shared the same pattern of elevation or reduction between the groups. ANOVA results clarified that these metabolites differed significantly between PD patients and HC, irrespective of the *LRRK2* mutation status. This underscores the potential relevance of stearic acid (C_18_H_36_O_2_), eicosanoic acid (C_20_H_40_O_2_), nonadecanoyl MG (C_22_H_44_O_4_), palmitic acid (C_16_H_32_O_2_), homophytanic acid (C_21_H_42_O_2_) in the context of PD, potentially serving as early disease biomarkers (Table 4). Additionally, monoglycerides (MGs) were significantly higher in non-manifesting *LRRK2* carriers (Table 4). An ANOVA confirmed these MGs also differed significantly between healthy individuals and PD patients. Notably, MG levels were not only highest in *LRRK2* patients, but all subgroups exhibited significantly higher levels compared to HC (Fig. 7). Moreover, octadecanoyl MG (C_21_H_42_O_4_) and nonadecanoyl MG (C_22_H_44_O_4_) emerged as highly relevant for differentiating PD patients from HC (Supplementary Fig. 2), suggesting MGs might be suitable candidates for early disease biomarkers, although the exploratory nature of this analysis, should be borne in mind. Additionally, the control group showed outliers with elevated values overlapping the range seen in the PD group, as illustrated in the boxplots (Fig. 5). This overlap and deviation within the control group raise the possibility that individuals in a prodromal stage of PD may have been inadvertently included among the controls. Future studies incorporating PD biomarkers such as CSF aSyn-SAA will help to elucidate the relationship between prodromal PD and early breathome changes.

Considering the presence of pathogenic variants within the PD patient group, this study presents specific metabolite patterns that enable the distinction of the subgroups (Supplementary Fig. 5-7). However, the classification of the subgroups based on metabolomic profiles in both body fluids was only partially accurate (Supplementary Fig. 7). Patients with pathogenic variants in *LRRK2* and *PRKN* genes in particular were misclassified, whereas those with a *GBA1* variant showed better results for both body fluids. This finding implies that *GBA1* mutations potentially lead to more distinctive metabolic changes compared to the other mutations. However, small sample sizes (12 with *LRRK2* and 6 with *PRKN* mutation) limit the results (Table 1A) and should be considered exploratory. Nevertheless, discernible differences exist between PD patients with and without pathogenic variants, as all IPD patients were correctly classified based on the metabolic pattern compared to patients with pathogenic variants (Supplementary Fig. 7). However, the small number of discernible metabolites identified in a volcano analysis (10 in exhaled breath and 23 in blood plasma analysis) (Supplementary Figs. 5 and 6) indicates that, as expected based on the many similarities between IPD and genetic PD, the distinctions are not as pronounced as when differentiating between PD patients and HC, wherein the volcano analysis identified over 80 metabolites.

While our study provides valuable insights into the metabolic profile in PD patients, certain limitations should be acknowledged to ensure accurate interpretation. The biggest challenge of this untargeted approach using high-resolution MS remains assigning identified masses to molecular structures^55^. However, while the proposed identities are putative, the indicated chemical formulas are accurate (mass error < 1 ppm). To confirm the proposed structures, the top metabolites will undergo MS/MS validation in future work. Limitations pertaining to our methodological approach have been addressed in our previous paper^30^. Furthermore, it should be noted that potential comorbidities in patients that could influence the metabolome were not taken into account. Control and covariate analyses focusing on comorbidities need detailed consideration. Another limitation of the study design is the inclusion of two independent control groups for breath and plasma analysis with different age ranges, showing potential imbalances between comparator groups. However, the independent control groups strengthened the validity of breath as a promising alternative biospecimen, as metabolite overlaps between PD patients and controls were observed in both specimens.

Although the control and PD groups for breath analysis differed in age range, potential confounding by age appears to have a minor impact on the observed metabolomic differences. This is supported by age-adjusted sensitivity analyses, where 7 out of 10 metabolites remained significantly different between groups. Future investigations should consider that the remaining three metabolites may be partially influenced by age-related effects. To strengthen these observations, it will be essential to replicate the findings in an age-matched cohort in future studies. Such validation would help confirm if the metabolites truly reflect disease-specific changes.

One additional constraint is the varied PD stages among patients, making it challenging to correlate metabolite profiles with PD progression. A more homogeneous patient population and additional clinical data are required to correlate metabolite patterns with symptom severity.

Although subgroup analyses revealed some metabolomic differences among PD patients carrying *LRRK2* or *PRKN* variants, these groups were small (*LRRK2* *n* = 12, *PRKN* *n* = 6) and therefore underpowered for definitive statistical inference. As such, these analyses are exploratory and intended to be hypothesis-generating. The inclusion of only four non-manifesting *LRRK2* variant carriers contributes to the associated uncertainty, and the wide intervals observed for several metabolites (Table 4) underscore the need for larger metabolomics studies to validate the proposed biomarker candidates. Conducting longitudinal studies is crucial for tracking individual metabolomic changes and determining if they later develop symptomatic conditions.

In conclusion, metabolomic profiles of blood plasma and exhaled breath effectively differentiated PD patients from HC, revealing distinct metabolomic patterns. Substantial differences were observed between these fluids, indicating that alternative matrices like breath add valuable information about (patho)physiological processes. Our untargeted metabolomic approach delineates a non-volatile metabolome of PD, laying the foundation for assessing clinical biomarkers in exhaled breath. The metabolomic breath profiling identified 7 significant metabolites, five of which were also detected in non-manifesting *LRRK2* carriers, suggesting a potential link to early PD development before clinical symptoms appear. Most of the proposed metabolites are intermediates in fatty acid metabolism, introducing new metabolites for breath analysis in PD. However, as the annotations are based solely on accurate mass and lack MS-MS confirmation, structural assignments remain putative. Future investigations should therefore validate their identities using a targeted MS/MS approach, clarify their role in PD pathophysiology and progression, and assess their utility as biomarkers for early diagnosis. This work evaluating differences in the breathome of PD patients represents an important initial step towards establishing a diagnostic breathprint, eventually paving the way for targeted and effective interventions.

## Methods

### Study design

By employing a previously established method^30^, this experimental study performed metabolomic profiling of exhaled breath of PD patients, both with and without pathogenic variants. Between July 2021 and August 2023, this project enrolled patients who were genetically characterized in either the LIPAD^56^ or ROPAD^6^ study, or both. The inclusion criteria required PD diagnosis according to the Movement Disorder Society (MDS) and participants aged at least 18^6,56^.

Additionally, the study population was subdivided based on the presence of pathogenic genetic variants. PD patients were subdivided into a group without a positive genetic testing report (idiopathic PD) and patients with genetic variants in the *LRRK2*, *PRKN*, and *GBA1* genes (mPD). Genetic testing of the included patients was primarily performed within the framework of two multicenter studies: ROPAD^6^ and SysMedPD^45^. In brief, most patients underwent gene panel sequencing. In a subset of patients, specific testing for *PRKN* or known variants in *LRRK2* and *GBA1* was performed; if positive, no further testing was conducted. All SysMedPD participants additionally underwent multiplex ligation-dependent probe amplification (MLPA) to detect copy number variants in the PRKN gene. Control individuals have not been genetically tested.

The *LRRK2* group comprised participants harboring one of the following *LRRK2* variants: p.Gly2019Ser (c.6055 G > A), and p.Tyr1699Cys (c.5096 A > G)^56^.

The *GBA1* group comprised participants with at least one of the following variants: p.Thr408Met (c.1223 C > T), p.Glu365Lys (c.1093 G > A), p.Asn409Ser (c.1226 A > G), p.Leu483Pro (c.1448 T > C), p.Gly241Arg (c.721 G > A), p.Arg398Gln (c.1193 G > A), p.Arg86* (c.256 C > T), p.Asn227Ser (c.680 A > G), p.Asp179His (c.535 G > C), p.Asp448His (c.1342 G > C), p.Glu427Lys (c.1279 G > A), and p.Leu363Pro (c.1088 T > C). The *PRKN*-variant carrier group included carriers of variants such as p.Arg275Trp (c.823 C > T), p.Cys253Tyr (c.758 G > A), p.Glu79* (c.336 G > T), p.Val56Glu (c.167 T > A), and p.Gly430Asp (c.1289 G > A), occurring either alone or in combination with *PRKN* copy number variants, including exon 2 or 3 duplication or exon 3, 4, 5 and/or 6 deletion. Supplementary Table 2 provides an overview of all identified variants and their occurrence within the subgroups.

Furthermore, to evaluate the potential of breath analysis, we used the commonly used diagnostic body fluid, blood plasma for comparison. Rather than directly comparing breath and blood plasma samples from the same individual, our objective was to assess the overall utility of breath analysis relative to blood sample analysis. To ensure an independent evaluation of the diseased sample groups, we included two distinct healthy control groups: one providing breath samples and the other providing blood plasma samples. Both groups consisted of healthy individuals without a PD diagnosis.

Additionally, healthy individuals with a first- or second-degree relative who tested positive for a genetic variant (*LRRK2*, *GBA1*) were also recruited. The inclusion criteria for the healthy breath control group were consistent with previous reports^30^

This study adhered to the principles outlined in the Declaration of Helsinki (as revised in 2013) and received ethical approval from the Ethics Committee of the University of Lübeck, Germany. Prior to participation, all patients gave informed consent. The methodological scheme of the study is presented in Supplementary Fig. 9.

### Specimen collection and sample preparation

Exhaled breath samples were collected non-invasively utilizing a device equipped with a polymeric electret filter (three replicates per participant). The sample acquisition and extraction of the electret filter was performed according to established protocols^30,57^. For mass analysis, the residue from each sample was re-suspended in 350 µl methanol/H_2_O (1:1, v/v) and subsequently diluted 1:20 with methanol/H_2_O (1:1 v/v) in 1.5 mL HPLC vials.

Plasma extraction from blood samples involved the collection of blood into two EDTA tubes, ensuring proper filling to maintain sample integrity. Following the collection, each tube was carefully inverted ten times to ensure thorough mixing. Subsequently, the tubes were immediately placed on ice to prevent protein degradation. The sample tubes were centrifuged at 2000 × *g* for 10 min at a temperature of 4 °C. After centrifugation, the resulting supernatant was transferred into a new 15 ml sterile polypropylene falcon tube and inverted to ensure homogeneity. The supernatant was then aliquoted into twelve 300 µl Cryovials, with each aliquot promptly frozen at −80 °C to preserve sample quality until further analysis.

Further sample preparation used 500 µL of the blood plasma. The plasma samples were extracted using a modified approach^58^, ensuring the efficient isolation and preparation of each sample component for further mass analysis. Extraction of the 500 µL blood samples resulted in a lipophilic and hydrophilic phase, as well as a protein pellet.

First, 500 µL methanol and 4 ml methyl *tert*-butyl ether (MTBE) were added to 500 µL of sample and vortexed. Samples were incubated at room temperature for 30 min using an overhead shaker with 25 rounds per minute (Trayster, Ika, Staufen, Germany). In the next step, 500 µL of ultra-pure water was added, and the mixture was then incubated for 10 min at room temperature without agitation. Lastly, all samples underwent centrifugation (Allegra X-30R/SX 4400, Beckman Coulter, Krefeld, Germany) at 1000 × *g* for 10 min.

The lipophilic phase was carefully transferred to a fresh 15 mL tube and stored at 4 °C for subsequent use. A second extraction cycle was conducted using the hydrophilic phase, following the same extraction steps as outlined before. Upon completion of the second extraction cycle, the lipophilic phases were combined and the hydrophilic phase was carefully transferred into a separate 15 mL tube, while the protein pellet was discarded.

The hydrophilic and lipophilic phases were dried using a vacuum concentrator (SpeedVac, Thermo Fisher Scientific, Bremen, Germany) and re-suspended, respectively. Thus, the hydrophilic and lipophilic phases were diluted at 1:1000 in the solvents methanol/H_2_O (1:1, v/v) and isopropanol/chloroform (3:1, v/v), respectively. The vials were stored at −80 °C until analysis *via* mass spectrometry.

### FT-ICR-MS measurements

This study utilized an ultra-high-resolution FT-ICR-MS system (7 Tesla, Solari-XR-X, Bruker, Bremen, Germany) coupled with an Infinity 1260 HPLC for direct sample injection (Agilent, Waldbronn, Germany). The mass spectrometric analysis was conducted according to our established method with identical parameters^30^.

Electrospray ionization was used in both positive and negative modes. Two different measurement methods detected ultra-small molecules and small molecules, achieving a detection range of 65–1500 m/z. The FT-ICR-MS featured a 2-omega cell with a precision of attained m/z signals to less than 1 ppm. The ICR-cell was calibrated utilizing sodium trifluoroacetate to achieve an accuracy of <0.5 ppm. Quality control samples, pooled from all samples, were prepared to assess sample stability and dilution^30,59^.

### Sample evaluation and data processing

The raw data was processed using the MetaboScape 2021b software (Bruker, Bremen, Germany). After importing, the dataset was recalibrated (accuracy <1 ppm) with a calibration list, containing >60 diverse matrix metabolites such as amino acids, carbohydrates or various lipids. The HMDB^31^ 2023 was used for metabolite annotation. Metabolites not included in the database were not identified in the dataset. In order to consolidate the dataset, further analysis steps comprised data normalization, duplicate removal, and data reduction of replicates.

First, the calculated bucket tables were exported to R 4.3, and all data was normalized using a PQN normalization script^60^. Consequently, the normalized data originating from the two measurement techniques as well as from both ionization modes were merged to create one single dataset covering the complete mass range (65–1500 m/z). Second, a method for duplicate removal was applied^60^. Accordingly, duplicate metabolites identified in both ionization techniques were eliminated by selecting the feature with the highest overall intensity across all samples for further analysis. Duplicates with lower scores were subsequently removed. The third and last step involved data reduction of the three replicates. To be qualified, a metabolite needed to be present in at least two of the three replicates obtained from the participants. Subsequently, the mean intensities of the replicates were calculated. In cases where a metabolite was detected in only one sample out of the three replicates, it was designated as “not detected” for that particular participant. Finally, the acquired dataset was used to delineate the non-volatile core metabolome of PD patients with and without pathogenic variants. Further, biostatistical analyses were applied to identify significant metabolites.

### Statistical analyses

The processed and evaluated data underwent biostatistical analyses using MetaboAnalyst 6.0^32^ with peak intensities and samples organized in columns (unpaired). A fixed value of 1/5 of the limit of detection was used to address the missing (zero) values. To identify the most stable subgroup-specific metabolites and reduce the potential impact of individual variations, further data processing and statistical analyses focused on metabolites detected in at least 10% of all samples. Statistical analysis applied median intensity values and the data used median normalization, log transformation, and Pareto scaling for normal distribution.

The metabolome analyses were performed employing MSEA with 1250 sets of metabolite-sub-classes. Metabolites present exclusively in healthy individuals but absent in PD patients were excluded prior to the MSEA to enhance the accuracy of our comparisons. Furthermore, univariate and multivariate analyses were combined to discover relevant metabolites in both breath and blood plasma. To compare two groups, such as PD patients and HC, we performed a volcano analysis. The fold-change was set to >1 (double effect size between the groups). The adjusted *p*-value was set to <0.1. This more permissive significance cutoff was chosen to retain metabolites that may occur at low concentrations or are close to the detection limit in exhaled breath. This approach ensures that potentially biologically relevant candidates are not excluded at this early discovery stage.

To enable comparisons between multiple groups, such as PD patients with different pathogenic variants, we applied a multiple ANOVA combined with a Tukey honestly significant difference (HSD) test for FDR correction. The significance threshold was set at a *p*-value of <0.05. In addition, a PLS-DA was performed to generate scores plot. This analysis identified the variable importance for projection (VIP) features, highlighting pattern differences between various subgroups.

Moreover, a classification analysis was conducted employing a random forest algorithm, integrated within the MetaboAnalyst 6.0^32^ software. The algorithm automatically picked random samples to generate a training dataset. Subsequently, a separate test dataset from the uploaded data was used to determine whether classification could be successfully performed based solely on metabolites. The seeds were selected randomly. Additionally, a maximum of 500 decision trees was permitted. The classification of two distinct subgroups was determined by both the OOB error and the classification error (class.error).

The significant metabolites obtained from the volcano analysis, PLS-DA and the random forest classification were combined in a Venn diagram, thereby evaluating the applicability of each test for analyzing the respective body fluid and identifying the most reliable metabolites across all three statistical approaches.

To evaluate the diagnostic power of particular metabolites, univariate and multivariate exploratory Receiver Operating Characteristic (ROC) analysis were executed with MetaboAnalyst Biomarker Analysis 6.0^32^. Herein, the PLS-DA and its built-in feature were selected for the classification and ranking methods, respectively. The analysis was conducted using two latent variables.

The ROC curves were generated applying Monte-Carlo cross-validation (MCCV) with balanced sub-sampling, e.g., an equal number of positive and negative cases. Specifically, each MCCV iteration involved randomly splitting the dataset into two parts. The training set used two-thirds of the samples to elaborate on the importance of the feature. The most important metabolite features (max. top 100) were subsequently used to build classification models. These models were validated on the remaining one-third of the samples (testing set). This process was repeated multiple times to calculate the performance and confidence interval of each model. Furthermore, classical univariate ROC analysis for single biomarkers used 500 bootstrappings for calculating the 95% confidence interval.

A sensitivity analysis was conducted to verify the robustness of the findings. For significant metabolites, an age- and gender-adjusted analysis was additionally performed to control for potential confounding by age and sex. Therefore, logistic regression predicting case-control status by age and the respective metabolite was performed. P-values of the regression coefficient of the metabolite were considered significant at a significance level of 0.05/10 = 0.5%.

## Supplementary information

Supplementary Information

## Data Availability

Data supporting the findings of this study are available within the paper and its Supplementary Information. Additional data are available from the corresponding authors on reasonable request.
